# Anti-Viral Activity of Bioactive Molecules of Silymarin against COVID-19 via In Silico Studies

**DOI:** 10.3390/ph16101479

**Published:** 2023-10-17

**Authors:** Chunye Zhang, Yuxiang Sui, Shuai Liu, Ming Yang

**Affiliations:** 1 Christopher S. Bond Life Sciences Center, University of Missouri, Columbia, MO 65212, USA; zhangcherryuniversity@gmail.com; 2 School of Life Science, Shanxi Normal University, Linfen 041004, China; sui998866@126.com; 3 The First Affiliated Hospital, Zhejiang University, Hangzhou 310006, China; liushuai202211@126.com; 4 Department of Surgery, University of Missouri, Columbia, MO 65212, USA; 5 NextGen Precision Health Institute, University of Missouri, Columbia, MO 65212, USA

**Keywords:** SARS-CoV-2, ACE2, silymarin, silymonin, silybin, anti-viral function, molecular docking, hub genes, immune regulation, drug-likeness

## Abstract

The severe acute respiratory syndrome-coronavirus-2 (SARS-CoV-2) infection drove the global coronavirus disease 2019 (COVID-19) pandemic, causing a huge loss of human life and a negative impact on economic development. It is an urgent necessity to explore potential drugs against viruses, such as SARS-CoV-2. Silymarin, a mixture of herb-derived polyphenolic flavonoids extracted from the milk thistle, possesses potent antioxidative, anti-apoptotic, and anti-inflammatory properties. Accumulating research studies have demonstrated the killing activity of silymarin against viruses, such as dengue virus, chikungunya virus, and hepatitis C virus. However, the anti-COVID-19 mechanisms of silymarin remain unclear. In this study, multiple disciplinary approaches and methodologies were applied to evaluate the potential mechanisms of silymarin as an anti-viral agent against SARS-CoV-2 infection. In silico approaches such as molecular docking, network pharmacology, and bioinformatic methods were incorporated to assess the ligand–protein binding properties and analyze the protein–protein interaction network. The DAVID database was used to analyze gene functions, such as the Kyoto Encyclopedia of Genes and Genomes (KEGG) pathway and Gene Ontology (GO) enrichment. TCMSP and GeneCards were used to identify drug target genes and COVID-19-related genes. Our results revealed that silymarin compounds, such as silybin A/B and silymonin, displayed triplicate functions against SARS-CoV-2 infection, including directly binding with human angiotensin-converting enzyme 2 (ACE2) to inhibit SARS-CoV-2 entry into the host cells, directly binding with viral proteins RdRp and helicase to inhibit viral replication and proliferation, and regulating host immune response to indirectly inhibit viral infection. Specifically, the targets of silymarin molecules in immune regulation were screened out, such as proinflammatory cytokines TNF and IL-6 and cell growth factors VEGFA and EGF. In addition, the molecular mechanism of drug-target protein interaction was investigated, including the binding pockets of drug molecules in human ACE2 and viral proteins, the formation of hydrogen bonds, hydrophobic interactions, and other drug–protein ligand interactions. Finally, the drug-likeness results of candidate molecules passed the criteria for drug screening. Overall, this study demonstrates the molecular mechanism of silymarin molecules against SARS-CoV-2 infection.

## 1. Introduction

**Figure 1:**
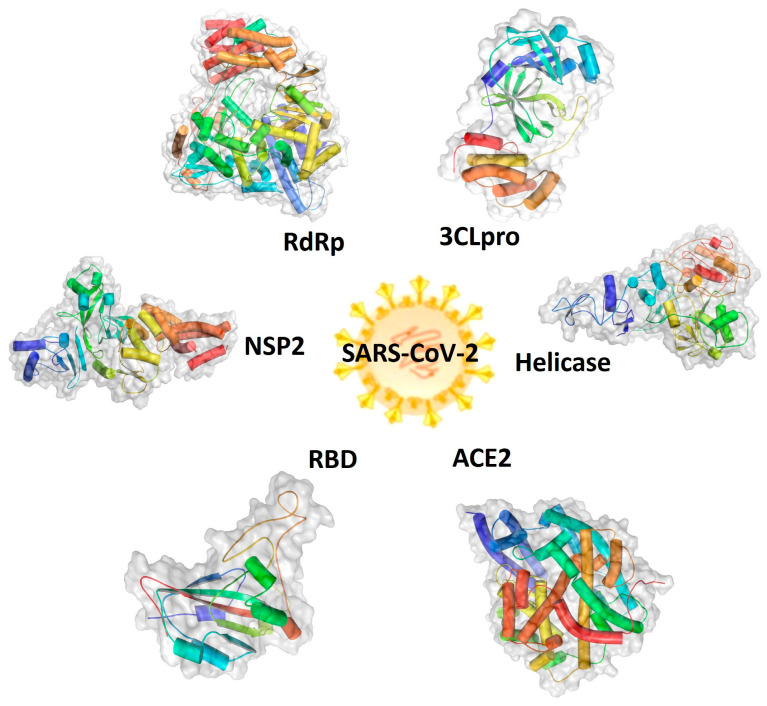
Graphic representations of the structures of druggable protein targets for the treatment of coronavirus disease 2019 (COVID-19). Therapeutic protein targets of severe acute respiratory syndrome coronavirus-2 (SARS-CoV-2) include the human angiotensin-converting enzyme 2 (ACE2) and the SARS-CoV-2 viral protein receptor-binding domain (RBD), helicase, nonstructural protein 2 (NSP2), 3C-like protease (3CLpro) or main protease (M), and RNA-dependent RNA polymerase (RdRp).

The severe acute respiratory syndrome-coronavirus-2 (SARS-CoV-2), a single-stranded, enveloped, positive-sense RNA virus, belongs to the genus of *Coronavirus*, the family *Coronaviridae*, and the order Nidovirales, and was the cause of the global coronavirus disease 2019 (COVID-19) pandemic [1,2,3]. The COVID-19 pandemic claimed many deaths worldwide in the last three years and led to a negative impact on economic development globally [4]. A rapid identification of therapeutic targets and agents is critical for fighting against the virus spread and preparing for its unpredictable re-emergence [5]. To date, the important viral targets include structured proteins, such as spike protein (S), envelope protein (E), membrane protein (M), and nucleocapsid (N) [6,7], and non-structured proteins (NSP1-NSP16), such as NSP2, NSP3 (3CLpro), and NSP13 (helicase). These viral proteins play essential roles in the virus, binding human angiotensin-converting enzyme 2 (hACE2) and allowing entry into cells, as well as virus replication, translation, and the formation of new viruses inside the host cells, functioning as potent druggable targets. The 3D structures of human ACE2 and SARS-CoV-2 components, including RNA-dependent RNA polymerase (RdRp), receptor-binding domain (RBD), NSP2, 3C-like protease (3CLpro) of NSP3, and NSP13 (helicase), are shown in Figure 1 [8,9,10,11,12,13]. Therefore, these proteins are attractive targets for the screening of small molecules as treatment agents for COVID-19 [3,14,15,16].

**Figure 2:**
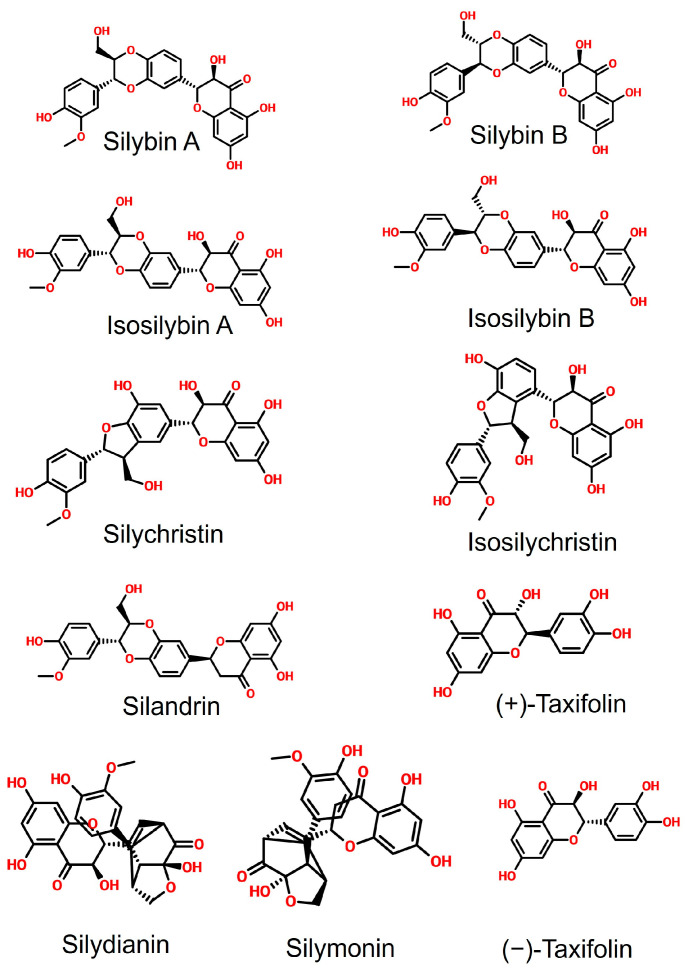
Chemical structure of silymarin components. These molecules include silybin A, silybin B, isosilybin A, isosilybin B, silychristin, isosilychristin, silandrin, (+)-taxifolin, silydianin, silymonin, and (−)-taxifolin.

The small molecules used as anti-viral agents can be mainly classified into three groups, including (1) de novo designed molecules, (2) re-proposed drug molecules approved for the treatment of other diseases, and (3) the components of natural products [17,18,19,20]. Silymarin, a mixture of herb-derived antioxidant polyphenolic flavonoids extracted from *Silybum marianum* L. (milk thistle), contains several bioactive compounds, including silybin A, silybin B, isosilybin A, isosilybin B, silychristin, isosilychristin, (+)-taxifolin, (−)-taxifolin, silydianin, silymonin, and silandrin (Figure 2) [21,22]. Silymarin is a well-known antioxidative, anti-apoptotic, and anti-inflammatory agent [23,24]. Previous research studies have demonstrated the anti-viral activity of silymarin against viruses, such as the dengue virus, the chikungunya virus (CHIKV), and the hepatitis C virus. Its anti-viral mechanisms include the direct binding with the dengue virus’ nonstructural protein 4B (NS4B), the inhibition of the RNA replication of CHIKV in a dose-dependent manner, and the downregulation of the CHIKV viral structural E2 protein expression, as well as the inhibition of influenza A virus (IAV) replication [25,26,27,28]. Moreover, silymarin also has an anti-inflammatory function, which can suppress the expression of proinflammatory cytokines such as interleukin 6 (IL-6) and tumor necrosis factor-alpha (TNF-α) and is closely associated with the regulation of expression of cytokines IL-2, IL-8, and IL-10, which are responsible for “cytokine storms” in COVID-19 patients [29,30,31,32]. Silymarin has been shown to have the potential effects to treat COVID-19 [33,34,35]. Therefore, we hypothesized that silymarin displays multiple anti-viral mechanisms against SARS-CoV-2 infection, including direct and indirect anti-viral activities. The underlying mechanisms of silymarin against SARS-CoV-2 infection could result from the direct anti-viral function and indirect immune modulation, such as inhibition of virus entry into host cells by blocking the host binding receptor or direct binding with the viral protein to inhibiting the replication and proliferation of the virus, as well as the modulation of host immune response. To date, the anti-viral effect of silymarin against SARS-CoV-2 has not been closely examined and related research is in demand [36,37,38]. Herein, as the objective of this study, we aimed to explore the druggable potential of silymarin compounds as anti-viral agents against SARS-CoV-2 infection and to examine the underlying molecular mechanisms of these molecules interacting with target proteins, as well as their immunoregulatory functions during anti-viral therapy.

To achieve our aims, we applied state-of-the-art approaches in this study by incorporating computer-aided drug discovery, network pharmacology, bioinformatic methods, and in silico approaches, such as molecular docking, to thoroughly investigate and examine the potential application of silymarin as an anti-viral agent against SARS-CoV-2 infection [39,40,41,42]. Computer-aided drug discovery is a powerful approach to accelerate drug screening, design, and discovery [43,44,45]. On one hand, it can be used to gain a better understanding of the properties of a target protein and to explore its potential as a therapeutic target [46,47]. On the other hand, molecular docking can be used to examine the binding affinity between candidate drugs and target proteins [48,49]. Meanwhile, the molecular mechanism of drug-target interaction can be determined [48]. Through the bioinformatic analysis of network pharmacology, immune-associated factors related to candidate drug compounds can be examined [41,50]. The pharmacological properties and drug-likeness of candidate compounds can also be thoroughly evaluated via these approaches [51,52,53].

Our results will provide data-based knowledge for the further validation, modification, and optimization of silymarin compounds as anti-viral agents against SARS-CoV-2 infection. This study also can inspire more upcoming research studies to investigate the immunomodulatory functions of these compounds, except their direct anti-viral activity.

## 2. Results

### 2.1. Molecular Docking between Target Proteins of SARS-CoV-2 and Silymarin Compounds

To examine the potential anti-viral drug candidates for SARS-CoV-2, we performed an intensive molecular docking analysis to evaluate the binding affinity between viral targets and silymarin molecules. Silymarin compounds included silybin A, silybin B, isosilybin A, isosilybin B, silychristin, isosilychristin, (+)-taxifolin, (−)-taxifolin, silydianin, silymonin, and silandrin, and the druggable target proteins of SARS-CoV-2 comprised RdRp, RBD, ACE2, NSP2, 3CL/NSP3, and helicase/NSP13. The results showed that among all the ligand–protein complexes, silymarin molecules displayed a higher binding affinity with human ACE2 compared to their binding ability with viral target proteins (Table 1). Among SARS-CoV-2 target proteins, the higher binding affinity was found in the complexes of helicase binding with silymonin (−9.7 kcal/mol), silybin B (−9.6 kcal/mol), silybin A (−9.5 kcal/mol), silandrin (−9.5 kcal/mol), and isosilybin B (−9.4 kcal/mol), and the complexes of NSP2 docking with silydianin (−9.3 kcal/mol), isosilybin A (−9.2 kcal/mol), silybin B (−9.1 kcal/mol), and silandrin (−9.1 kcal/mol), as well as the complexes of RdRp interacting with silymonin (−9.7 kcal/mol), silychristin (−9.3 kcal/mol), and silydianin (−9.2 kcal/mol), respectively (Table 1).

**Figure 3:**
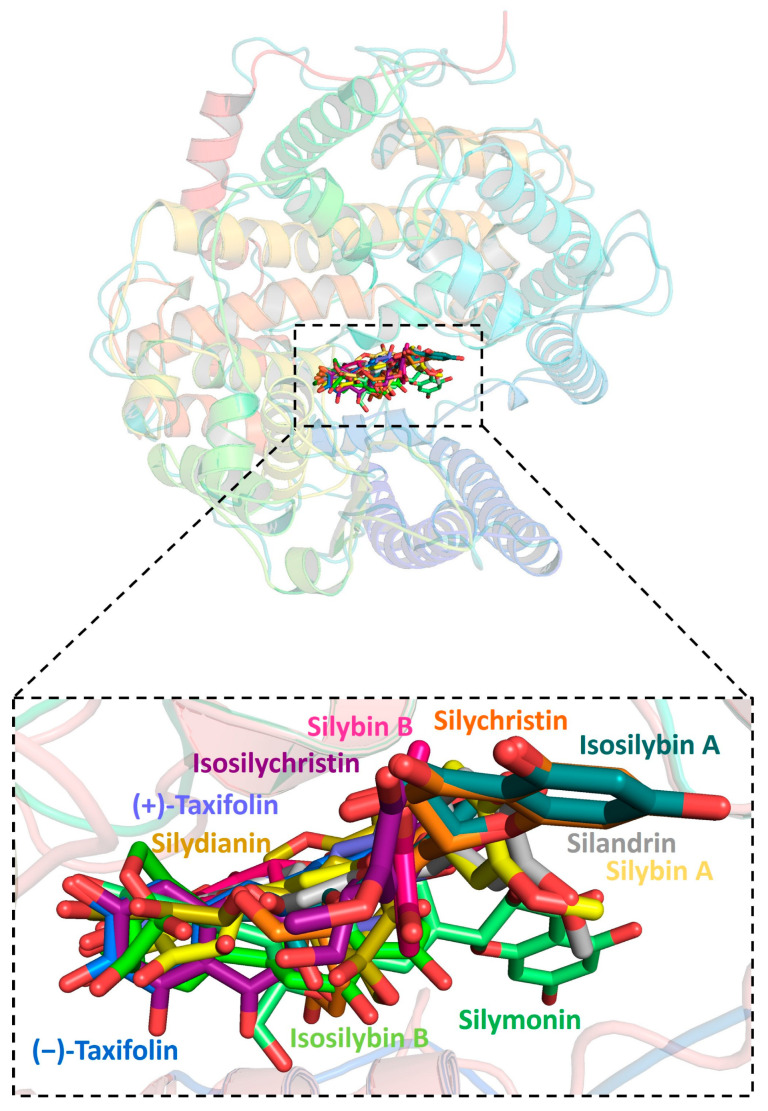
Graphic depiction of the binding site between human ACE2 and 11 molecules of silymarin.

**Figure 4:**
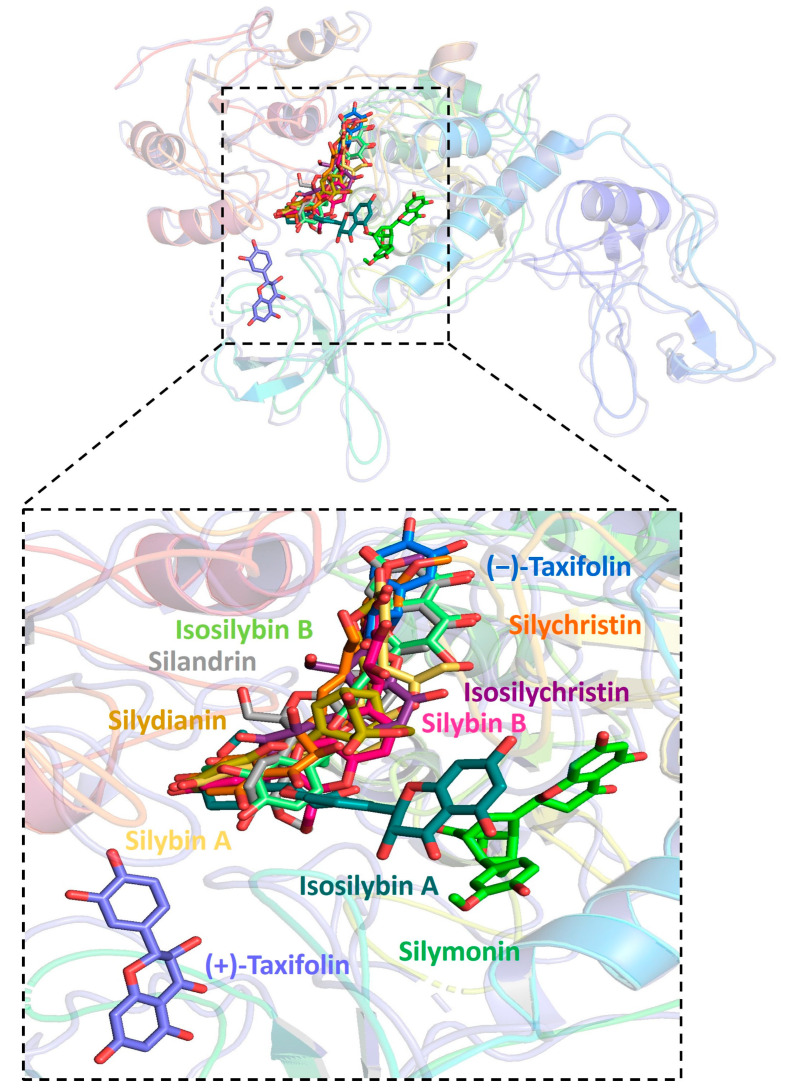
Graphic depiction of the binding site between SARS-CoV-2 helicase and 11 molecules of silymarin.

**Figure 5:**
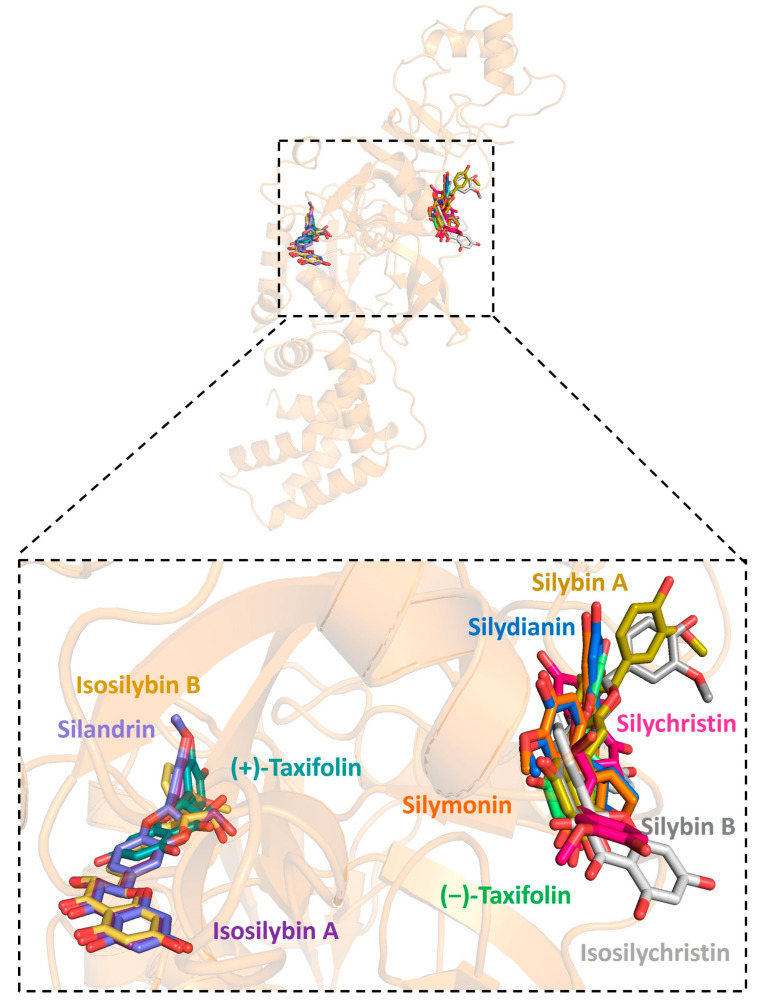
Graphic depiction of the binding site between SARS-CoV-2 NSP2 and 11 molecules of silymarin.

**Figure 6:**
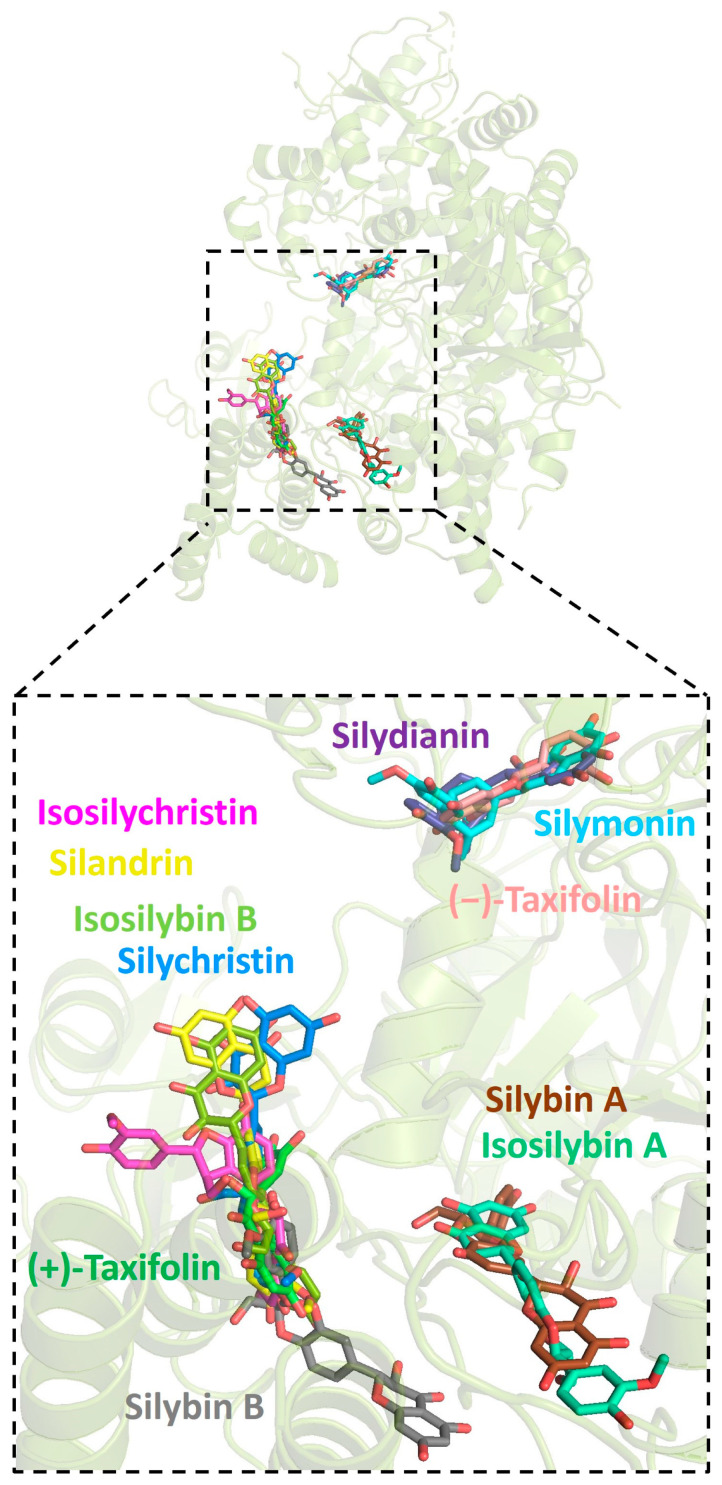
Graphic depiction of the binding site between SARS-CoV-2 RdRp and 11 molecules of silymarin.

Further analysis showed that all silymarin molecules were located in the same binding pocket when they were forming complex with human ACE2, shown in a figure (Figure 3). The binding pocket of silymarin molecules with viral helicase was also mainly located in the same major position with a slight difference for molecules isosilybin A, silymonin, and (+)-taxifolin (Figure 4). There are two major binding pockets for silymarin molecules binding with viral NSP2 (Figure 5). Three binding positions were shown for silymarin molecules binding with viral RdRp (Figure 6).

### 2.2. The Formation of Hydrogen Bonds in the Complex of Molecules and Host ACE2

**Figure 7:**
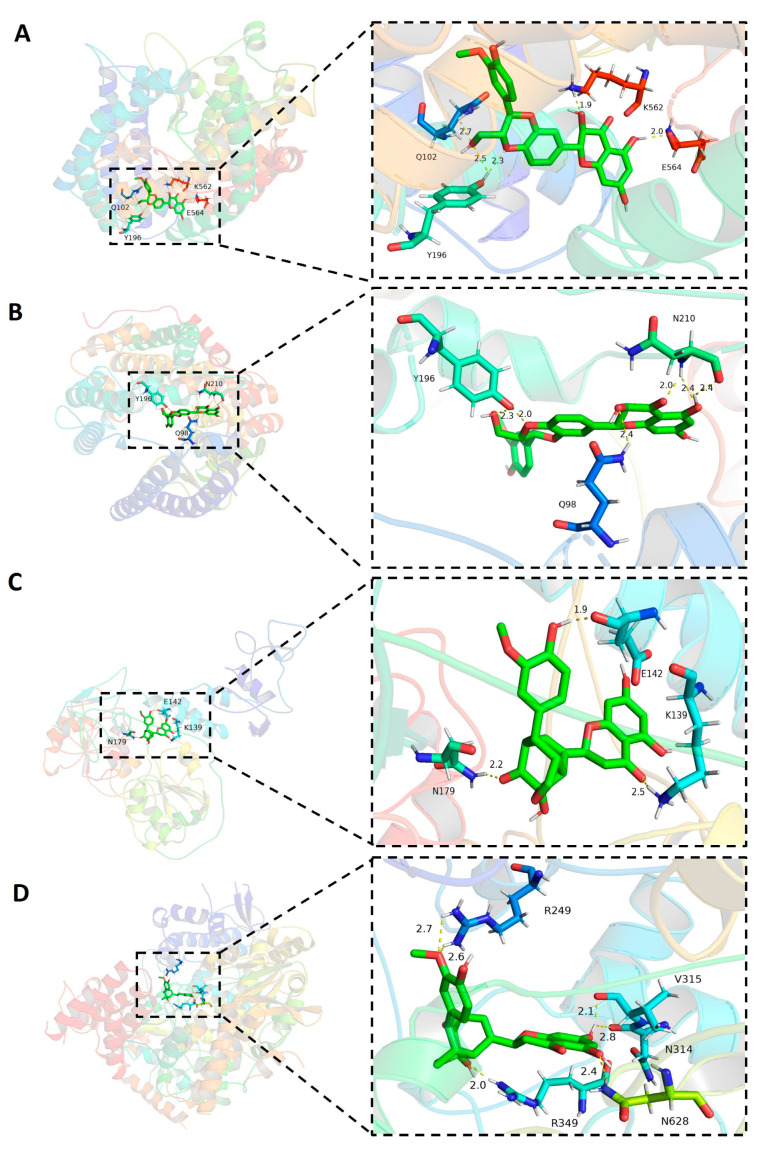
Hydrogen bonds and distances of small molecules in complex with target proteins. (**A**) Silybin A in complex with human ACE2. (**B**) Silybin B in complex with human ACE2. (**C**) Silymonin in complex with SARS-CoV-2 helicase. (**D**) Silymonin in complex with SARS-CoV-2 RdRp.

To explore the molecular interaction between candidate molecules and host ACE2, we chose the ligand–protein complexes with the highest binding affinity for further analysis. Among all silymarin molecules, molecules silybin A and silybin B had the higher binding affinity (−10.2 kcal/mol for both) with human ACE2 compared to other molecules. Hydrogen bonds (H-bonds) are important molecular interaction structures. In the complex of silybin A and ACE2, five H-bonds were formed between ligand silybin A and four residues Y196 (2 H-bonds), Q102, K562, and E564 (Figure 7A) of human ACE2. There were six H-bonds formed in the complex of molecule silybin B with human ACE2. Three residues of human ACE2 protein were involved in the formation of H-bonds (Figure 7B), including Y196 (2 H-bonds), N210 (3 H-bonds), and Q98 (1 H-bond). These H-bonds contributed to protein–ligand interaction in the binding complexes.

### 2.3. The Hydrogen Bonds Formed between Silymarin Molecules and Viral Helicase and RdRp

Molecule silymonin displayed higher binding affinity in complex with viral proteins helicase (−9.7 kcal/mol) and RdRp (−9.7 kcal/mol) compared to other molecules when binding with the rest of SARS-CoV-2 proteins (Table 1). Therefore, the H-bonds formed between silymonin and helicase or RdRp were analyzed. The results indicated that there were three H-bonds formed in the binding complex of silymonin and helicase, which were contributed by residues E142, K139, and N179 of viral protein (Figure 7C). In contrast, there were six hydrogen bonds formed in the silymonin-RdRp complex, which were contributed by R249 (2 H-bonds), R349 (1 H-bond), N314 (1 H-bond), V315 (1 H-bond), and N628 (1 H-bond) of viral protein (Figure 7D). These H-bonds contributed to the molecular interaction of silymonin with viral proteins helicase and RdRp.

### 2.4. Residues Involved in Hydrophobic and Other Protein–Ligand Interactions

**Figure 8:**
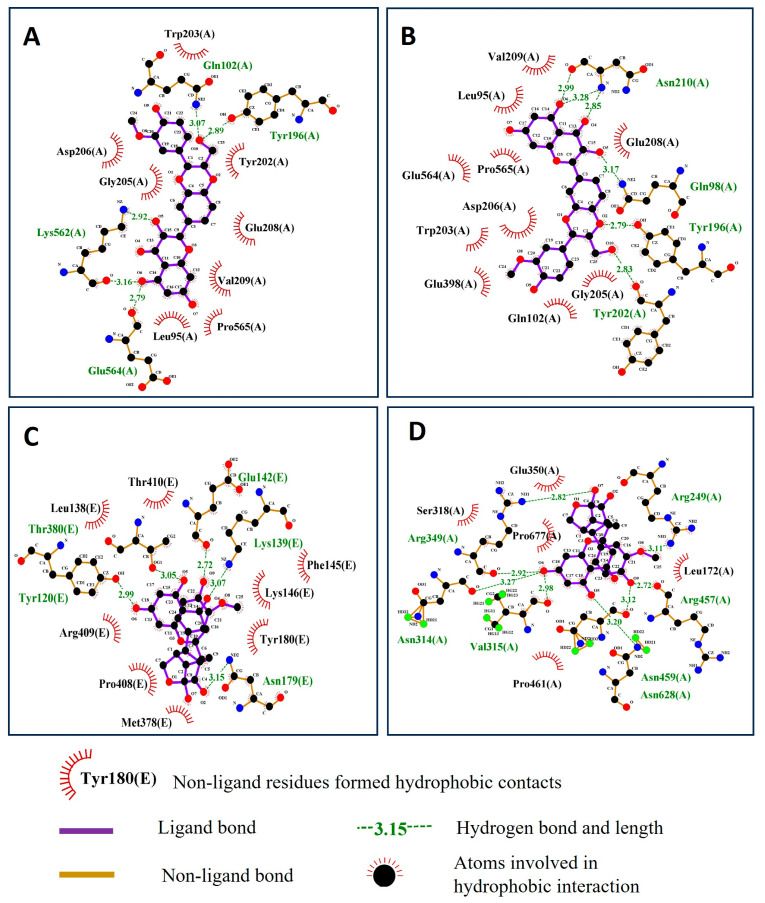
The residues that contribute to the hydrophobic interaction between ligands and target proteins. (**A**) Silybin A in complex with human ACE2. (**B**) Silybin B in complex with human ACE2. (**C**) Silymonin complex with SARS-CoV-2 helicase. (**D**) Silymonin in complex with SARS-CoV-2 RdRp. Red, black, and blue dots represent carbon, oxygen, and nitrogen atoms, respectively.

In addition to hydrogen bonds, hydrophobic interactions also contribute to the formation of the protein–ligand complex. The residues involved in the hydrophobic interactions for the complexes of silybin A and silybin B with human ACE2, as well as the complexes of silymonin with viral proteins helicase and RdRp, were analyzed and are shown in Figure 8 through 2D protein–ligand interactions.

**Figure 9:**
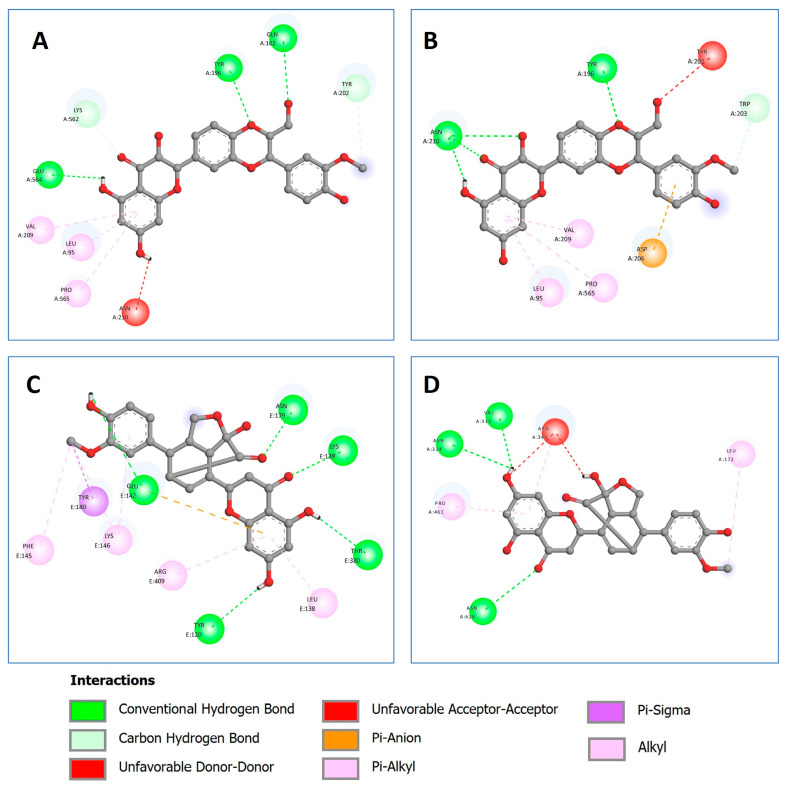
Other ligand–protein interactions. (**A**) Silybin A in complex with human ACE2. (**B**) Silybin B in complex with human ACE2. (**C**) Silymonin in complex with SARS-CoV-2 helicase. (**D**) Silymonin in complex with SARS-CoV-2 RdRp.

Other protein–ligand interactions were also shown in the silybin A-ACE2 complex, such as the carbon–hydrogen bond and pi-Alkyl (Figure 9A), and in the silybin B-ACE2 complex, such as the carbon–hydrogen bond, Pi-Alkyl, and Pi-Anion (Figure 9B). Meanwhile, other interactions in the silymonin–helicase complex were caused by Alkyl, Pi-Alkyl, Pi-Anion, and Pi-Sigma (Figure 9C), and the Alkyl and Pi-Alkyl interactions were also identified in the silymonin–RdRp complex (Figure 9D).

### 2.5. MM/GBSA and MM/PBSA Free Energy Calculation Results

To further validate the interaction of the complexes, MM/GBSA and MM/PBSA were applied for the free energy calculation with the ligand–molecule binding complexes. The calculation methods were based on the molecular dynamic simulations of the receptor–ligand complex [54]. In this study, we conducted both MM/GBSA and MM/PBSA free energy calculations using the fastDRH platform (Table 2) [55]. When the calculation was performed using MM/GBSA, the results revealed that the total binding free energies (PBTOT/GBTOT) for the screened complexes were ACE2–silybin A (−46.26 kcal/mol), ACE2–silybin B (−38.23 kcal/mol), helicase–silymonin (−38.73 kcal/mol), and RdRp–silymonin (−33.9 kcal/mol), respectively. When the calculation was performed using MM/PBSA, the results of the total binding free energies for the screened complex were ACE2–silybin A (−43.92 kcal/mol), ACE2–silybin B (−35.99 kcal/mol), helicase–silymonin (−35.81 kcal/mol), and RdRp–silymonin (−35.9 kcal/mol), respectively.

### 2.6. Drug-likeness Evaluation

To further assess the drug-likeness of those compounds, we applied the commonly used tool in drug discovery, SwissADME, to evaluate the pharmacokinetics and drug-likeness according to the Lipinski filter applied by Pfizer. The results suggested that silymarin compounds met the drug-likeness requirements based on the criteria of the Lipinski filter (Table 3). Although both silychristin and isosilychristin each had one violation, based on the statement “no more than one violation in general”, they also met the drug-likeness criteria.

### 2.7. Screening of COVID-19-Associated Genes and Silymarin Target Genes

The above-demonstrated investigation focused on the direct molecular interaction between silymarin molecules and human ACE2 protein or SARS-CoV-2 proteins, which contributed to the inhibition of virus binding, replication, proliferation, and infection in host cells. In addition, the immunoregulatory functions of candidate molecules were also explored in the subsequent study.

**Figure 10:**
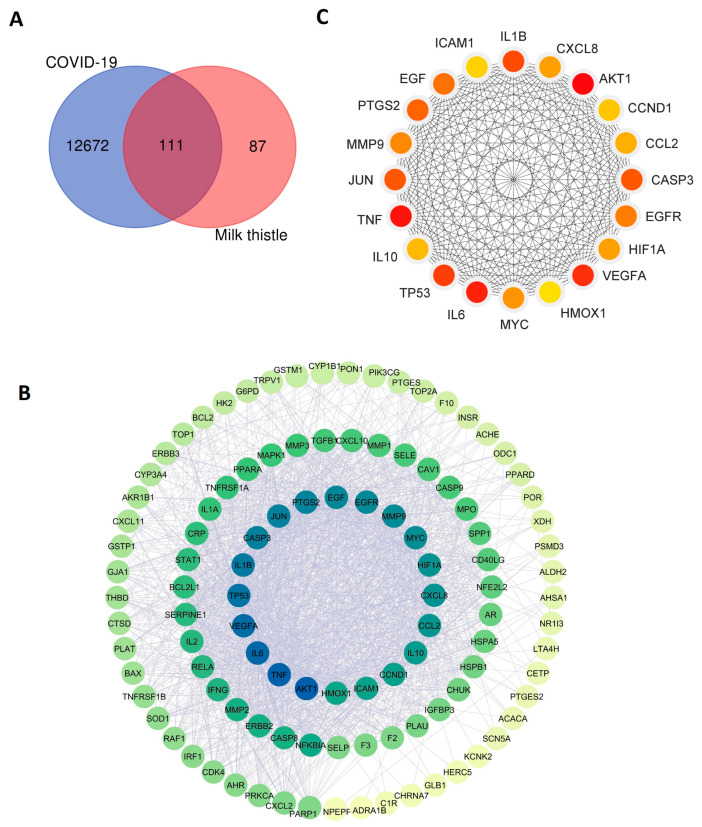
Analysis of COVID-19-associated genes and target genes of silymarin. (**A**) Venn diagram of shared genes between COVID-19-associated genes and target genes of silymarin (milk thistle). (**B**) Construction of protein–protein interaction network of the identified shared 111 genes. (**C**) Identification of 20 hub genes in the constructed PPI network.

First, we utilized bioinformatics tools and databases to identify COVID-19-associated genes. In total, 12,783 genes associated with COVID-19 were obtained from the human gene database GeneCards. Meanwhile, we retrieved 198 silymarin (milk thistle)-related target genes from the Traditional Chinese Medicine Systems Pharmacology Database and Analysis Platform (TCMSP) version 2.3. By comparing the two databases, 111 shared common genes between COVID-19-associated genes and drug target genes were identified (Figure 10A).

### 2.8. Construction of Protein–Protein Interaction Network of Shared Genes

To evaluate whether the identified 111 shared genes’ encoded proteins had biological connections and interactions, we constructed the protein–protein interaction (PPI) network by analyzing these 111 proteins using the bioinformatics tool STRING (Figure 10B). The results showed that the input 111 proteins formed 110 nodes and 1909 edges, with an average node degree of 34.7 and an average local clustering coefficient value of 0.689. The PPI enrichment *p*-value was less than 1.0e-16, which indicated that the input network has significantly more interactions than expected. These results suggest that the identified 111 proteins have more interactions among themselves, and they are biologically connected as a group rather than a random set of proteins that are purely based on the similarity of protein size and degree of distribution from the genome database. This biologically connected network provides a foundation for the further analysis of the hub genes within the network.

### 2.9. Identification of Hub Gene among the Shared Genes

Since the proteins encoded by shared 111 genes have biological connections as a group, we then performed the subsequent analysis to identify the hub genes from them within the network. This allowed us to easily visualize the functions of these shared genes. The top 20 hub genes were identified and ranked using the degree method, namely *AKT1*, *TNF*, *IL6*, *VEGFA*, *TP53*, *IL1B*, *CASP3*, *JUN*, *PTGS2*, *EGF*, *EGFR*, *MMP9*, *MYC*, *HIF1A*, *CXCL8*, *CCL2*, *IL10*, *CCND1*, *ICAM1*, and *HMOX1* (listed from high to low score) (Figure 10C). The identified 20 hub genes play a significantly central role in the shared gene set.

### 2.10. Kyoto Encyclopedia of Genes and Genomes (KEGG) Analysis of 111 Shared Genes

**Figure 11:**
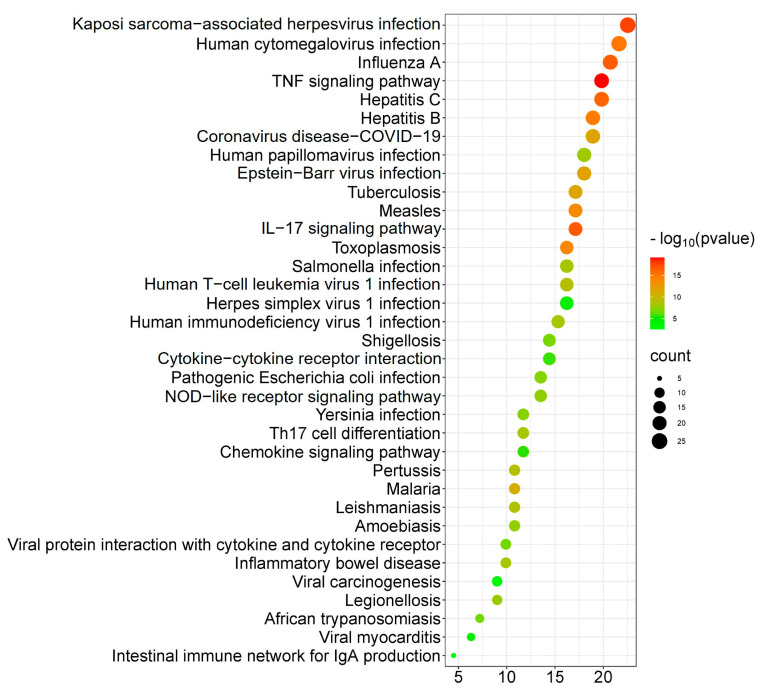
Analysis of Kyoto Encyclopedia of Genes and Genomes (KEGG) enrichment. The top 35 signaling pathways related to viral infection were identified from the shared 111 genes.

To further explore how the shared 111 genes were involved in disease and molecular interactions, we analyzed the Kyoto Encyclopedia of Genes and Genomes (KEGG). The results showed that those genes were highly enriched and involved in viral infection disease, host immune response, and inflammation signaling pathways (Figure 11). For example, the signaling pathways involved in virus infection were identified, including the coronavirus disease COVID-19 and other viral infections, such as Kaposi sarcoma-associated herpesvirus infection, human cytomegalovirus infection, influenza A, hepatitis C/B, Epstein–Barr virus infection, and human papillomavirus infection. In addition, these genes were involved in signaling pathways associated with host immune response and inflammation, such as the TNF signaling pathway, the IL-17 signaling pathway, the Th17 cell differentiation, cytokine–cytokine receptor interactions, the chemokine signaling pathway, the nucleotide-binding and oligomerization domain (NOD)-like receptor signaling pathway, and the intestinal immune network for Immunoglobulin A (IgA) production. These results demonstrate that silymarin molecules may display both direct anti-viral activity and indirect immunoregulatory functions against SARS-CoV-2 infection, contributing to two important functions against viral infection.

### 2.11. Gene Ontology (GO) Functional Enrichment Analysis of 111 Shared Genes

**Figure 12:**
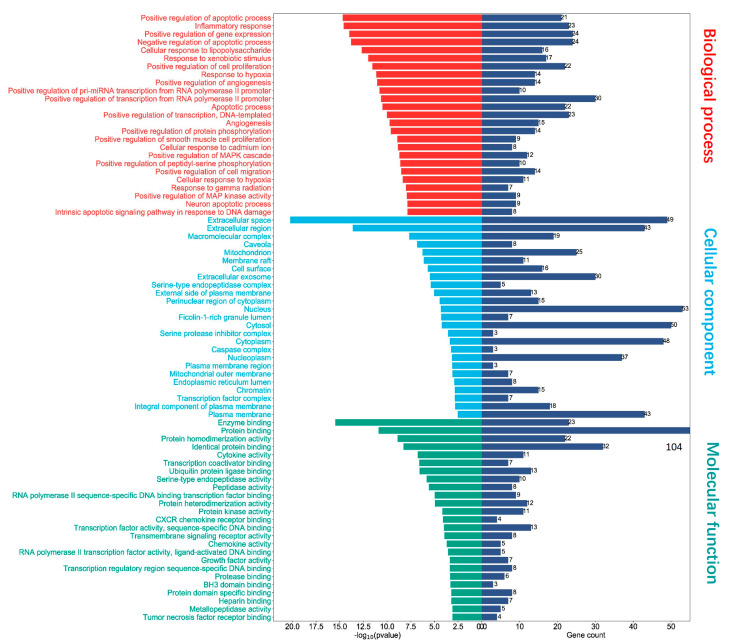
Gene Ontology (GO) enrichment analysis. Top 25 of each category including molecular function, cellular component, and biological process of the 111 shared genes. Blue column represents the number of genes involved in molecular function, cellular component, or biological process.

To illustrate the biological relevance and contribution of those shared 111 genes in biological process (BP), cellular component (CC), and molecular function (MF), we performed the Gene Ontology (GO) analysis. As presented in Figure 12, the results depicted the activity of those genes in biological processes, such as apoptotic process, inflammatory response, cellular response to lipopolysaccharide, response to xenobiotic stimulus, and cell proliferation and migration. The functions of these genes involved in cellular components included extracellular space, macromolecular complex, mitochondrion, membrane raft, cell surface, extracellular exosome, and the external side of the plasma membrane. Most importantly, those genes in particular contributed to several molecular functions, such as enzyme binding, protein binding, cytokine activity, peptidase activity, RNA polymerase II transcription factor activity, CXCR chemokine receptor binding, chemokine activity, transmembrane signaling receptor activity, growth factor activity, heparin-binding, and tumor necrosis factor receptor binding. The above-mentioned processes and activities are critical for COVID-19 disease infection and host cell response.

## 3. Discussion

The COVID-19 pandemic is caused by the infection of SARS-CoV-2, resulting in a tremendous loss of lives globally during the outbreak [56]. The negative effect caused by the disease remains and significant effort is required to combat post-pandemic era issues [57,58,59,60]. Thus, the urgent necessity for the exploration of anti-viral strategies for newly emerged viruses is critical for both outbreaks of COVID-19 and other coronavirus diseases [61,62,63]. Drug discovery is a time-consuming process and has a huge cost. Currently, fast and effective strategies to combat viral infection include vaccine development, drug-repurposing, and the exploration of natural products. The drug properties and safety or potential side effects of repurposed drugs and natural products have been already investigated, testified, or illustrated by previous animal studies and clinical trials or applications. This renders the timesaving and cost–efficacy benefits in drug discovery [64,65,66,67]. Upon the emergence of the COVID-19 disease, extensive computational techniques like virtual screening, molecular docking, molecular dynamics simulation, and the Molecular Mechanics Poisson–Boltzmann Surface Area (MM/PBSA) approach for calculating binding free energy were applied to investigate and explore the potential drug molecules against SARS-CoV-2 infection by targeting PLpro, main protease, RdRp, etc. [68,69,70,71]. Significant progress has been made using computer-aided drug discovery strategy as a powerful tool to accelerate the drug screening processes.

Silymarin is a mixture of natural products consisting of flavonolignans and flavonoid ingredients, which are extracted from the natural plant *Silybum marianum*, also known as *Milk thistle*. The major bioactive components of silymarin include a mixture of flavonolignans, such as silybin, isosilybin, silychristin, isosilychristin, silydianin, silymonin, silandrin, and flavonoid taxifolin. For a long time, they have been well-known as anti-oxidative, anti-inflammatory, and antifibrotic agents, with immunomodulatory functions, and their medical functions have been examined and studied in many diseases [72,73]. For instance, studies have demonstrated that silymarin and its derives have hepatoprotective functions via different molecular mechanisms, such as a reduction in high fructose-induced oxidative stress and the modulation of peroxisome proliferator-activated receptor (PPAR)-α activation, TNF-α expression, and phosphatidylinositol-3-kinase (PI3K)/Akt (protein kinase B)/mammalian target of rapamycin (mTOR) signaling pathways [74,75,76,77]. The hepatoprotective functions were also shown in the reduction in liver damage caused by an infection of hepatitis C virus (HCV) and hepatitis B virus (HBV) [25]. The potent anti-viral efficiency of silymarin has been demonstrated by many studies. The anti-viral functions of active silymarin compounds are mediated by different mechanisms against different viruses, such as to inhibit dengue virus (DENV) infection by binding viral NS4B [26], to suppress viral replication and protein synthesis of CHIKV [27,78], to inhibit viral replication and ROS induction of the Mayaro virus [79], to suppress the viral RNA synthesis and replication of influenza viruses [80], and to regulate T-cell activation, proliferation, and metabolism in order to inhibit human immunodeficiency virus infection [81].

Given the diverse anti-viral functions of silymarin, the anti-viral mechanism of bioactive silymarin compounds against SARS-CoV-2 should be studied in the pandemic and post-pandemic eras. Thus, in this study, we employed the commonly used drug discovery strategies, such as molecular docking, to comprehensively examine the binding affinity of candidate bioactive molecules of silymarin with a series of viral proteins and human ACE2 acting as receptors for the entry of viruses into host cells. Additionally, considering the syndrome of the “cytokine storm” in patients with COVID-19 and the anti-inflammatory function of silymarin, we further investigated the immunoregulatory function of candidate compounds. We incorporated network pharmacology to analyze drug target genes, the shared parts with the genes associated with COVID-19, and the involved signaling pathways of the shared genes. Next, we examined the anti-viral activity of silymarin compounds and the underlying mechanisms, including the binding activity with host ACE2, direct interaction with viral proteins, and immunoregulatory functions.

Firstly, we found that silymarin bioactive compounds had high binding affinities with viral host receptor ACE2 (Table 1). Human ACE2 is expressed in many cell surfaces (e.g., heart and lungs), which is recognized and well-known as the binding receptor of SARS-CoV-2, mediating virus entry into the host cells to trigger virus replication and proliferation cycle [82,83]. The intervention of virus-ACE2 receptor binding can reduce the entry of viruses to host cells to decrease the subsequent viral infection. Therefore, targeting ACE2 is one of the best strategies for exploring treatment agents against SARS-CoV-2 infection [84,85]. Our study showed that silymarin compounds have a higher binding affinity with ACE2 compared to viral proteins. Among these compounds, silybin A and silybin B showed the highest binding affinity (−10.2 kcal/mol for both) in complexes with ACE2 (Table 1).

Secondly, in addition to the direct binding with host ACE2, we also examined the direct binding affinity of silymarin compounds with a series of virus therapeutic target proteins, which play a critical role in virus replication and proliferation [86,87]. The results illustrated the higher binding affinity between silymonin and virus RdRp (−9.7 kcal/mol) and between silymonin with helicase (−9.7 kcal/mol). Silybin A (−9.5 kcal/mol) and silybin B (−9.6 kcal/mol) also showed high binding affinity when docking with helicase. These results suggest that molecules silybin A, silybin B, and silymonin are bioactive agents with potent therapeutic functions against SARS-CoV-2 infection. The MM/GBSA and MM/PBSA free energy calculations are widely used approaches for evaluating ligand–protein binding interactions. The calculation is based on molecular dynamic simulations, and the successful application of MM/GBSA and MM/PBSA methods has been demonstrated in different systems [54]. In this study, we utilized these methods to calculate the MM/GBSA and MM/PBSA free energies to further evaluate the screened complexes of ACE2–silybin A, ACE2–silybin B, silymonin–helicase, and silymonin–RdRp. The results (Table 2) further show that those compounds could serve as potent anti-viral drug candidates.

Third, most importantly, with the network pharmacology analysis of the shared genes between COVID-19-associated genes and drug target genes, we found the potent immunomodulatory function of silymarin compounds against COVID-19. The identified top 20 hub genes from 111 shared genes, such as *TNF*, *IL6*, *IL1B*, *CXCL8*, *CCL2*, and *IL10* (Figure 10B), play a significant role in the signaling pathways of immune regulation (Figure 11). The increased levels of inflammatory cytokines, such as IL-1, IL-2, IL-4, IL-6, IL-8, IL-10, IL-13, IL-17, and TNF-α, were detected in patients with SARS-CoV-2 infection compared to control subjects in the clinic [88,89,90,91,92]. TNF is a major factor that promotes inflammation [93,94]. IL-6, IL-1b, and CCL2 belong to the member of pro-inflammatory cytokines [95,96,97,98]. IL-10 is a cytokine with inflammation suppression activity and promotes the cytolytic activity of T cells [99,100]. The bioactive molecules of silymarin can also target those cytokines (Figure 10B,C). Given the anti-inflammatory and immunomodulatory functions of silymarin, it can play essential roles in modulating the syndrome of “cytokine storm” in COVID-19 patients [33,35].

Notably, candidate silymarin compounds, such as silybin A/B and silymonin, display triplicate functions against SARS-CoV-2 infection, including directly binding with the host receptor ACE2 to inhibit SARS-CoV-2 entry to the host cells, directly binding with viral proteins RdRp and helicase to inhibit virus replication and proliferation, and regulating host immune response to indirectly inhibit viral infection.

Moreover, the drug-likeness of these drug candidates was also examined based on the criteria adopted from Pfizer for drug discovery screening (Table 3). The results allow the further optimization and investigation of using the candidate drugs as therapeutic agents in future studies to enhance their pharmacological efficiency and reduce their side effects. Even though computer-aided drug discovery is a powerful tool that can be used to accelerate the speed of candidate drug exploration, the challenges and limitations also need to be considered. Therefore, experimental validation such as in vitro studies using 2D and 3D cell culture models (e.g., organoids) and in vivo experiments in animal models and clinical trials are required for the further verification of those potential therapeutic agents [34,35,71]. The synergetic treatment efficacy conferred by silymarin compounds should be evaluated as part of the validation comparison with appropriate positive controls, for which in vitro and in vivo studies are required.

## 4. Methods and Materials 

### 4.1. Source of Target Proteins and Small Molecules for Molecular Docking

The crystal structures of proteins used for molecular docking were obtained from the PDB database and were prepared well for the docking program. These were ACE2 (PDB ID: 6M0J, Chain A), RBD (PDB ID: 6M0J, Chain E), helicase (PDB ID: 7CYQ, Chain E), NSP2 (PDB ID: 7MSX), RdRp (PDB ID: 6XQB, Chain A), and 3CLpro (PDB ID: 6LU7, Chain A). Small molecules were obtained from the PubChem public database and were well prepared for the docking program. The open-source program AutoDock Vina v.1.2.0 employed by Cavity Detection-Guided Blind Docking (CB-DOCK) was used for conducting the molecular docking [101].

The well-prepared proteins (e.g., water was removed and hydrogens were added) and ligands (with polar hydrogens and Gasteiger charges) were submitted to CB-DOCK2. Template ligands were initially based on a high topological similarity (FP2 ≥ 0.4), followed by the calculation and comparison of queried proteins and selected ligand–protein complexes. The sequence identity >40% and pocket RMSD ≤ 4Å were used as parameters for subsequent cavity identification and docking. The latest version of Auto Dock Vina (1.2.0) was employed by the platform to select the best binding site and pose for molecular docking, and the parameters were used as default.

Polar hydrogens and Gasteiger charges were automatically added to ligands by the adopted program. Essential hydrogen atoms and Kollman charges were added to the proteins [68]. The detected major cavity volume and center point axis of the proteins were ACE2 (cavity volume 377, center_x: −30.131, center_y: 32.105, center_z: −21.789), RBD (cavity volume 112, center_x: −26.146, center_y: 30.75, center_z: 32.165), Helicase (cavity volume 4521, center_x: 237.31, center_y: 180.593, center_z: 158.891), NSP2 (cavity volume 597, center_x: 108.984, center_y: 111.533, center_z: 105.009), RdRp (cavity volume 5147, center_x: 107.948, center_y: 103.779, center_z: 110.47), and 3CLpro (cavity volume 258, center_x: −13.676, center_y: 11.294, center_z: 71.716). No specific site was defined for the grid box in the blind docking procedure, and the whole protein was enclosed within the docking range. According to the binding affinity, the complex with the lowest binding energy was identified. The binding affinity was calculated using a scoring function that was adopted by AutoDock Vina, including the intermolecular energy (ligand–receptor) and the intramolecular energy (ligand–ligand).

### 4.2. Interaction Analysis of the Protein–Ligand Complex

The ligand–protein binding affinity results and binding mode were screened based on the docking scores from the docking program. The analysis and visualization of the ligand–protein binding interaction were prepared and presented using Pymol software. The 2D interaction analysis and presentation of the hydrogen bonds and other interactions were performed using PDBsum [102] and Discovery Studio Visualizer v21.1.0.20298.

### 4.3. Pharmacokinetics and Drug-likeness Evaluation

The SwissADME bioinformatics tool is one of the commonly used tools in drug discovery for examining the pharmacokinetic properties and drug-likeness of molecules. In the current study, SwissADME was applied for the examination of the pharmacokinetics and drug-likeness of the selected silymarin molecules [103].

### 4.4. MM/GBSA and MM/PBSA Free Energy Calculation

The fastDRH web platform was used to conduct the free energy calculation usingthe Molecular Mechanics/Generalized Born Surface Area (MM/GBSA) and the Molecular Mechanics/Poisson–Boltzmann Surface Area (MM/PBSA) [54,55]. The molecular mechanics (MM) force fields for protein and ligand were ff19SB (with OPC water model) and GAFF2, respectively. The pose re-scoring procedure of GB1 (radii = mbondi, γ = 0.00720, β = 0.0000) was used. For truncation radius, default parameters on the platform were performed for both pose re-scoring and hotspot prediction.

### 4.5. Identification of Drug Target-Related Genes and COVID-19 Disease-Related Genes

Traditional Chinese Medicine System Pharmacology Database (TCSMP) was used for analyzing the silymarin (milk thistle) target genes. The data were filtered based on the value for good pharmacological activity, including oral bioavailability (OB) ≥ 30% and drug-likeness (DL) ≥ 0.18) [104,105,106]. Based on this filter criteria, the related target gene names of silymarin compounds were further retrieved and converted to official gene symbols. The duplicated genes were removed. GeneCards database (www.genecards.org, accessed on 2 July 2023) was used for analyzing COVID-19-associated genes [107,108]. The queried keyword “COVID-19” was used to screen the COVID-19-related genes and the official gene symbols were retrieved from the database. The duplicated genes were cleaned.

### 4.6. Protein–Protein Interaction (PPI) Network Construction

We applied a bioinformatic database STRING (Search Tool for the Retrieval of Interacting Genes/Proteins) (http://string-db.org/, accessed on 2 July 2023) for the construction of a protein–protein interaction (PPI) network. This tool allowed the construction of a network based on the functional interactions and associations of a set of queried proteins. The information source adopted by the STRING database was obtained from both experiments and online databases. The functional-related crossing interaction of multiple queried proteins was analyzed [109]. For this study, the result was analyzed specifically for “Homo sapiens”, and 0.4 was set as the threshold for a confidence score of protein interactions, and it was considered significant for the minimum required interaction. The other parameters were selected as default values.

### 4.7. Cytoscape for Network Analysis

Bioinformatics tool Cytoscape platform (https://cytoscape.org/, accessed on 2 July 2023) was applied for analyzing and visualizing complex networks. The topological characteristic ‘‘degree” was selected for the calculation of the central attribute of hub nodes among the networks. Other parameters were set as the default values. The results included the connecting nodes and molecular interactions, as well as the identification of key hub genes from the network of shared genes between COVID-19-associated genes and silymarin target genes [110].

### 4.8. Analysis of the KEGG Signaling Pathway and GO Functional Enrichment

The Gene Ontology (GO) analysis and Kyoto Encyclopedia of Genes and Genomes (KEGG) analysis were performed to assess the functional potential of shared genes between COVID-19 disease-associated genes and drug target genes. The functional enrichment including a biological process (BP), cellular component (CC), molecular function (MF), and signaling pathway enrichment of shared genes were further studied. The database for the Annotation, Visualization, and Integrated Discovery (DAVID) system was used to analyze the results [111,112].

## 5. Conclusions

The computational study and bioinformatic network pharmacology analysis provided effective ways to speed up the drug screening process and generate preliminary data for evaluating the efficacy of candidate silymarin compounds as anti-SARS-CoV-2 infection drugs. This computer-based approach also validates the molecular mechanism of drug–target interactions. However, both in vitro and in vivo studies are required to further evaluate the therapeutic efficacy of these drug candidates against SARS-CoV-2 infection, which are the limitations of the current study.

## Figures and Tables

**Table 1:** Molecular docking results of silymarin molecules and human ACE2 and SARS-CoV-2 proteins (kcal/mol).

Target Molecules	Human	SARS-CoV-2				
	ACE2	RBD	Helicase	NSP2	RdRp	3CLpro
Silybin A	−10.2	−7.6	−9.5	−8.9	−8.9	−8
Silybin B	−10.2	−7.9	−9.6	−9.1	−8.9	−8.6
Isosilybin A	−10.0	−7.5	−8.8	−9.2	−8.8	−8.5
Isosilybin B	−9.5	−8.2	−9.4	−8.9	−8.9	−8.7
Silychristin	−9.6	−7.6	−9.1	−8.8	−9.3	−8.4
Isosilychristin	−9.2	−7.1	−8.9	−8.3	−8.4	−8.6
Taxifolin (+)	−8.6	−6.9	−7.9	−7.7	−8	−7.2
Taxifolin (−)	−8.0	−6.8	−7.5	−7.9	−7.9	−7.6
Silydianin	−9.5	−8.8	−9.2	−9.3	−9.2	−8.5
Silymonin	−9.9	−8.6	−9.7	−9	−9.7	−8.4
Silandrin	−9.7	−8.3	−9.5	−9.1	−8.9	−8.3

**Table 2:** MM/PB(GB)SA scores (kcal/mol) for the protein–ligand binding complexes.

MM/GBSA (per Residue Binding Energy Decomposition on a Protein–Ligand Complex)									
Complex	ELE	VDW	INT	GAS	PBSUR/GBSUR	PBCAL/GB	PBSOL/GBSOL	PBELE/GBELE	PBTOT/GBTOT
ACE2–Silybin A	0	−55.64	0	−55.64	−6.58	15.96	9.37	15.96	−46.26
ACE2–Silybin B	0	−46.35	0	−46.35	−6.3	14.42	8.12	14.42	−38.23
Helicase– Silymonin	0	−45.96	0	−45.95	−6.26	13.49	7.23	13.49	−38.73
RdRp–Silymonin	0	−39.32	−0.04	−39.36	−4.59	10.05	5.46	10.05	−33.9
**MM/PBSA (per Residue Binding Energy Decomposition on a Protein–Ligand Complex)**									
**Complex**	**ELE**	**VDW**	**INT**	**GAS**	**PBSUR/GBSUR**	**PBCAL/GB**	**PBSOL/GBSOL**	**PBELE/GBELE**	**PBTOT/GBTOT**
ACE2–Silybin A	0	−51.98	0	−51.99	−7.11	15.17	8.06	15.17	−43.92
ACE2–Silybin B	0	−45.42	0	−45.41	−6.03	15.45	9.42	15.45	−35.99
Helicase– Silymonin	0	−39.01	0	−39.01	−5.11	8.3	3.2	8.3	−35.81
RdRp–Silymonin	0	−42.81	0	−42.81	−5.6	12.52	6.91	12.52	−35.9

Abbreviations: ELE, electrostatic energy as calculated by the MM force field; VDW, van der Waals contribution from MM; INT, internal energy arising from bond, angle, and dihedral terms in the MM force field; GAS, total gas phase energy (sum of ELE, VDW, and INT); PBSUR/GBSUR, non-polar contribution to the solvation-free energy calculated by an empirical model; PBCAL/GB, the electrostatic contribution to the solvation-free energy calculated by PB or GB, respectively; PBSOL/GBSOL, sum of non-polar and polar contributions to solvation; PBELE/GBELE, sum of the electrostatic solvation free energy and MM electrostatic energy; PBTOT/GBTOT, final estimated binding free energy (kcal/mol) calculated from the terms above.

**Table 3:** Pharmacokinetic and drug-likeness assessment results.

Lipinski Filter (Pfizer)	Physicochemical Properties			Lipophilicity	Drug-likeness	Drug-likeness
	MW ≤ 500	N or O ≤ 10	NH or OH ≤ 5	MLOGP ≤ 4.15	Lipinski #violations	Yes or No
SilybinA	482.44	10	5	−0.4	0	Yes
SilybinB	482.44	10	5	−0.4	0	Yes
IsosilybinA	482.44	10	5	−0.4	0	Yes
IsosilybinB	482.44	10	5	−0.4	0	Yes
Silychristin	482.44	10	6	−0.4	1	Yes
Taxifolin (+)	304.25	7	5	−0.64	0	Yes
Taxifolin (−)	304.25	7	5	−0.64	0	Yes
Silydianin	482.44	10	5	−0.45	0	Yes
Silymonin	466.44	9	4	0.32	0	Yes
Silandrin	466.44	9	4	0.37	0	Yes
Isosilychristin	482.44	10	6	−0.4	1	Yes

## Data Availability

All the data supporting reported results can be found in this study.

## Abbreviations

COVID-19	Coronavirus disease 2019
SARS-CoV-2	Severe acute respiratory syndrome coronavirus 2
NSP	Nonstructural protein
ACE2	Angiotensin-converting enzyme 2
hACE2	Human angiotensin-converting enzyme 2
RdRp	RNA-dependent RNA polymerase
RBD	Receptor-binding domain
3CLpro	3C-like protease (3CL) or main protease (M)
MW	Molecular weight
KEGG	Kyoto Encyclopedia of genes and genomes
GO	Gene Ontology
PPI	Protein–protein interaction
BP	Biological process
CC	Cellular compound
MF	Molecular function
IL-6	Interleukin 6
TNF-α	Tumor necrosis factor-alpha
CXCR	Chemokine receptor
ROS	Reactive oxygen species
AKT1	AKT serine/threonine kinase 1
VEGFA	Vascular endothelial growth factor A
TP53	Tumor protein 53
CASP3	Caspase-3
JUN	Transcription factor Jun
PTGS2	Prostaglandin-endoperoxide synthase 2
EGF	Epidermal growth factor
EGFR	Epidermal growth factor receptor
MMP9	Matrix metallopeptidase 9
MYC	MYC proto-oncogene
HIF1A	Hypoxia-inducible factor 1-alpha
CXCL8	Chemokine ligand 8
CCL2	Chemokine ligand 2
CCND1	Cyclin D1
ICAM1	Intercellular Adhesion Molecule 1 (known as Cluster of Differentiation 54:CD54)
HMOX1	Heme oxygenase 1 gene
IgA	Immunoglobulin A
NOD	Nucleotide-binding and oligomerization domain
